# Aminostyrylbenzofuran Directly Reduces Oligomeric Amyloid-β and Reverses Cognitive Deficits in Alzheimer Transgenic Mice

**DOI:** 10.1371/journal.pone.0095733

**Published:** 2014-04-23

**Authors:** Sang-Hyun Lee, YoungSoo Kim, Hye Yun Kim, Young Hoon Kim, Maeng Sup Kim, Jae Yang Kong, Mun-Han Lee, Dong Jin Kim, Young Gil Ahn

**Affiliations:** 1 Hanmi Research Center, Hanmi Pharmaceutical Co., Ltd., Hwaseong-si, Gyonggi-do, Republic of Korea; 2 Department of Biochemistry, College of Veterinary Medicine, Seoul National University, Seoul, Republic of Korea; 3 Center for Neuro-Medicine, Brain Science Institute, Korea Institute of Science and Technology, Seoul, Republic of Korea; 4 Biological Chemistry Program, University of Science and Technology (UST), Daejeon, Republic of Korea; 5 Department of Biochemistry and Biomedical Sciences, Seoul National University, College of Medicine, Seoul, Republic of Korea; 6 College of Pharmacy, Keimyung University, Daegu, Republic of Korea; Boston University School of Medicine, United States of America

## Abstract

Alzheimer's disease is an irreversible neurodegenerative disorder that is characterized by the abnormal aggregation of amyloid-β into neurotoxic oligomers and plaques. Although many disease-modifying molecules are currently in Alzheimer clinical trials, a small molecule that inhibits amyloid-β aggregation and ameliorates the disorder has not been approved to date. Herein, we report the effects of a potent small molecule, 6-methoxy-2-(4-dimethylaminostyryl) benzofuran (KMS88009), that directly disrupts amyloid-β oligomerization, preserving cognitive behavior when used prophylactically and reversing declines in cognitive behavior when used therapeutically. KMS88009 exhibited excellent pharmacokinetic profiles with extensive brain uptake and a high level of safety. When orally administered before and after the onset of Alzheimer's disease symptoms, KMS88009 significantly reduced assembly of amyloid-β oligomers and improved cognitive behaviors in the APP/PS1 double transgenic mouse model. The unique dual mode of action indicates that KMS88009 may be a powerful therapeutic candidate for the treatment of Alzheimer's disease.

## Introduction

Alzheimer's disease (AD) is a progressive neurodegenerative disorder that is characterized by the conformational transition of amyloid-β (Aβ) into soluble oligomers, protofibrils and fibrils, which accumulate to form insoluble plaques during the abnormal aggregation process. The presence of misfolded Aβ species is highly correlated with the severity of the neuroinflammation following neurotoxicity and is a direct cause of the neurodegeneration in AD [1], [2]. Thus, numerous anti-aggregation therapeutic strategies have been proposed, such as the use of Aβ aggregation inhibitors and Aβ production-reducing molecules [3], [4]. However, harmful preformed Aβ aggregates remain in the brain after these molecules have retarded further amyloidogenesis and/or reduced Aβ production. The persistence of these aggregates is important because they accumulate in AD brains long before the onset of mild cognitive impairment, which is the initial stage of AD [5], [6]. Therefore, the clearance of Aβ aggregates is considered the most effective treatment for AD [1], [4], [7]. To date, no drug has been developed that can simultaneously inhibit and reverse toxic Aβ aggregation as well as subsequently ameliorate the abnormal behaviors associated with AD.

In our previous study, we synthesized a series of anti-amyloidogenic aminostyrylbenzofuran derivatives by introducing a styryl conjugated system consisting of *E*,*E*-1-iodo-2,5-bis-(3-hydroxycarbonyl-4-methoxy)-styrylbenzene and curcumin onto a benzofuran nucleus [8]. Herein, we describe one of these molecules, 6-methoxy-2-(4-dimethylaminostyryl) benzofuran (KMS88009), which has an excellent pharmacokinetic profile and significant anti-amyloidogenic effects that ameliorate cognitive impairment in the APP/PS1 double transgenic (TG) mouse model of AD [9]. In the present study, the anti-amyloidogenic small molecule KMS88009 was evaluated to determine its physicochemical properties, pharmacokinetics and toxicity prior to behavioral tests and post-mortem analysis of APP/PS1 TG mice.

## Materials and Methods

### Materials

KMS88009, 6-methoxy-2-(4-dimethylaminostyryl) benzofuran, was synthesized as previously described [8]. All other materials were obtained from commercial sources and were the highest grade available.

### Animals

Specific pathogen-free male CD-1 (ICR) (9-week old, male, 30±5 g) mice and Spraue-Dawley (SD) rats (9-week old, male, 265±25 g) were obtained from Orient Bio (Korea). Beagle dog (7-month old, male, 9±1 kg) were obtained from WooJung BSC (Korea). Cynomolgus monkeys (36-month old, male, 2.3±2 kg) were obtained from Guangxi Research Center of Primate Laboratory Animal (China). Animals were acclimated for 7 days prior to studies initiation with an evaluation of health status. APP/PS1 TG mice (strain name: B6C3-TG (APPswe, PS 1dE9) 85Dbo/J) were obtained from Jackson Laboratory (Bar Harbor, Maine, USA). APP/PS1 TG mice begin to develop Aβ plaques at 6–7 months of age and memory deficits at 7 month of age or older [10]. Throughout the experiments, animals were housed in a controlled environment [temperature 23±2°C, humidity 50±10%, light period: 6:00 AM to 6:00 PM (PK analyses) or 6:00 PM to 6:00 AM (efficacy analyses)]. Each monkey was maintained in a cage (700 W×800 L×670 H cm, 2 columns, 1 row, Squeeze Back type with stainless steel) per 1 head. For monkeys, Toys (kong toy, mirrors and feeding puzzle) and vitamin candy were supplied. The attending veterinarian monitored their health. For all animals, food and water were available *ad libitum*. All animals were randomized into one of groups in each study.

### Ethics statement

All animal experiments were carried out in accordance with the National Institutes of Health guide for the care and use of laboratory animals (NIH Publications No. 8023, revised 1978). All animal procedures used for this study were prospectively reviewed and approved by the Animal Care and Use Committee of the Hanmi Research Center. All procedures, including the monkey study, were carried out under anesthesia and all efforts were made to minimize the number of animals and suffering. For monkeys, we monitored changes of body weight, hair, eyes/nose, movement and posture and scored 0 (normal) to 3 (significant alternations) to refine the humane endpoints. If total score of symptomatic signs was higher than 8 points, the study was terminated. If more than 2 individual criteria reached the maximum points of 3, the study was terminated. As a method of euthanasia, monkeys were anesthetized with pre-administration of Ketamin (Yuhan Corp., Korea) and then with heavy dose of Pentobarbital (Hanlim Pharm Co., Ltd, Korea).

### Metabolic stability

We performed *in vitro* metabolic stability experiments using liver microsomes (LM) of various species (mouse, rat, dog, monkey and human LM (BD Gentest, USA)) under the following condition for drug-LM mixtures: KMS88009 (5 µM), microsomes (1 mg/mL) and NADPH regeneration solution (1.3 mM NADP^+^, 3.3 mM glucose-6-phosphate, 3.3 mM MgCl_2_ and 0.4 U/mL glucose-6-phosphate dehydrogenase) in 50 mM potassium phosphate buffer solution. The mixtures were preincubated at 37°C for 10 min and the metabolism reaction (in triplicate) was initiated by addition of KMS88009 to the incubation mixture. At 0, 5, 15, 30 and 60 min, 50 µL aliquots of the reaction mixtures were quenched using 100 µL acetonitrile as a stop solution. These samples were centrifuged for 5 min at 13,000 rpm and the supernatant was analyzed using HPLC. The microsomal activity was monitored using verapamil or testosterone (Sigma-Aldrich, USA) as positive controls. To determine the half-life (T_1/2_), the analytic peak areas were converted to percent drug remaining, using the T = 0 peak area values as 100%. Percent of remaining compounds was calculated compared to the initial quantity at 0 time point.

### CYP inhibition

Cytochrome P450 (CYP) inhibition assay was carried out according to the principle of the Crespi assay. Fluorescent probe substrates (BD Gentest, USA) used for each CYP isozyme were: 3-cyano-7-ethoxycoumarin for CYP1A2 and CYP2C19, 7-benzyloxy-trifluoromethylcoumarin for CYP3A4, 7-methoxy-4-trifluoromethylcoumarin for CYP2C9 and 3-[2-(N,N-diethyl-N-methylamino)ethyl]-7-methoxy-4-methyl-coumarin for CYP2D6. The IC_50_ for each isozyme by KMS88009 was determined using a 5-point concentration (0.62, 1.85, 5.56, 16.67 and 50 µM) curve with 3-fold serial dilution. Known CYP inhibitors (BD Gentest), ketoconazole (0.1, 0.2, 0.6, 1.7 and 5 µM, for CYP3A4), furafylline (1.2, 3.7, 11.1, 33.3 and 100 µM, for CYP1A2), sulfaphenazole (0.1, 0.4, 1.1, 3.3 and 10 µM, for CYP2C9), tranylcypromine (1.2, 3.7, 11.1, 33.3 and 100 µM, for CYP2C19) and quinidine (0.006, 0.02, 0.06, 0.17 and 0.5 µM, for CYP2D6) were used as positive controls. The plate was incubated at 37°C for 10 min before the addition of enzyme-substrate mixture (100 µL) and sample plates were incubated for 15 min (CYP1A2), 30 min (CYP3A4, 2C19 and 2D6) and 45 min (CYP2C9) on a shaker at 37°C. All reactions were stopped by addition of 75 µL of 0.1 M tris in acetonitrile and analyzed by an LC-MS/MS product ion monitoring method on Agilent 1200 HPLC (Agilent, USA) and API-5000 mass spectrometer (Canada). IC_50_ value was calculated using SigmaPlot 10.0 (Systat Software Inc., USA).

### Single dose toxicity studies

KMS88009 was orally administered by gavage at dose levels of 0 (vehicle), 1,000 and 2,000 mg/kg. Each group consisted of 3 rats. Vehicle was DMSO/tween-20/saline (3.3∶3.3∶93.4) mixture. Administration volume of vehicle and KMS88009 was 10 mL/kg. Mortality, physical abnormalities and signs of pain/distress were daily observed in all animals. Body weight of each animal was measured before the administration and on the 1^st^, 4^th^, 8^th^, 11^th^ and 14^th^ days.

### Repeated oral dose toxicity studies

KMS88009 was orally administered once a day by gavage for 14 consecutive days at dose level of 0 (vehicle), 100, 300 and 1,000 mg/kg (n = 5 per group). Changes of body weight, blood and organ weight were observed with mortality and symptomatic signs. Organs were collected at necropsy; adrenal, kidney, thymus, spleen, heart, lung, liver, testis and brain. The absolute organ weights were measured and the organs-to-body weight ratios were calculated. Blood samples for hematological evaluation were collected from vein of animals under ether anesthesia. Food was withheld overnight before necropsy and blood collections. EDTA was used as an anticoagulant for hematology samples. White blood cell (WBC), red blood cell (RBC), hemoglobin concentration (HGB), hematocrit (HCT), mean corpuscular volume (MCV), mean corpuscular hemoglobin (MCH), mean corpuscular hemoglobin concentration (MCHC), cell hemoglobin concentration mean (CHCM), cell hemoglobin (CH), red cell distribution width (RDW), hemoglobin distribution width (HDW), platelet count (PLT), mean platelet volume (MPV) and reticulocyte were measured by a hematological autoanalyzer (ADVIA 120, Bayer, USA). To get the serum for biochemical analyses, blood samples in a separation tube were centrifuged at 4,000 rpm for 10 min on the day of necropsy. The serum was kept frozen at -70°C until analysis. Sodium citrate was used as an anticoagulant for biochemical analyses. Serum biochemical parameters including aspartate aminotransferase (AST), alanine aminotransferase (ALT), alkaline phosphatase (ALP), total bilirubin (T-Bil), albumin, total protein (TP), albumin/globulin ratio (A/G ratio), glucose, total cholesterol (CHO), triglyceride (TG), lactate dehydrogenase (LDH), creatine kinase (CK), blood urea nitrogen (BUN), creatinine (CRE), blood urea nitrogen/creatinine ratio (B/C ratio), Na^+^, K^+^ and Cl^-^ were evaluated by an autoanalyzer (Shimadzu CL-7200, Shimadzu Co., Japan).

All data for single and 2-week repeated oral dose toxicity studies were expressed as the mean ± SD Difference between control and treated groups were evaluated with one-way analysis of variance (ANOVA) followed by Dunnett's test. Value *P*<0.05 was taken as being statistically significant. Statistical analyses were performed using GraphPad Prism software (USA).

### Pharmacokinetics in mice, rats, dogs and monkeys

Pharmacokinetics profile of KMS88009 was obtained using ICR mice, SD rats, beagle dogs and cynomolgus monkeys. Animals were fasted overnight before KMS88009 administration. Mice were divided into 2 groups to receive KMS88009 as a single dose of 2.1 mg/kg intravenous (IV) bolus or 10 mg/kg orally (PO) by gavage. Additionally, KMS88009 was administered orally to estimate the BBB penetration in mice (10, 30 and 100 mg/kg). In rat, KMS88009 was administrated as an IV bolus (1.25 mg/kg) via the tail vein or by oral gavage (2.5 mg/kg). Dog received KMS88009 either as an IV bolus (1 mg/kg) via the great saphenous vein or by oral gavage (4 mg/kg). KMS88009 was administrated to monkeys as an IV bolus (1 mg/kg) via the femoral vein or by oral gavage (4 mg/kg). Vehicle for IV and PO administration was DMSO/tween-20/saline (3.3∶19.8∶79.6). All animals (n = 3 per group) were sacrificed and concentration of KMS88009 in plasma and brain were measured at 1/12, 1/4, 1/2, 1, 2, 4, 10 and 24 hr after IV administration and at 1/2, 1, 2, 4, 10 and 24 hr after PO administration. Brain samples were weighed and Daunce-homogenized in 10-fold volume of ice-chilled 4% bovine serum albumin (BSA) solution at room temperature. Plasma and brain homogenates were kept frozen at −70°C until analysis. The levels of KMS88009 were determined by the LC-MS/MS analysis. Pharmacokinetic parameters, time to the maximum concentration (T_max_), maximum concentration (C_max_), elimination half-life (T_1/2_), volume of distribution (V_d_), clearance (Cl) and mean residual time (MRT), of KMS88009 were calculated based on plasma concentration-time data by a non-compartmental method using WinNonlin Version 5.2 (Pharsight, USA). Area under the curve from 0 to 24 hr (AUC_0-24h_) and area under the curve from 0 to infinity (AUC_INF_) were obtained by linear and log linear trapezoidal summations.

### Learning and memory behavior tests

Drugs (KMS88009 and scyllo-inositol) were orally (PO) administered once a day until the end of behavior tests. An aggregation inhibitor Scyllo-inositol was administered as a control. In the prophylactic trial, APP/PS1 TG mice were treated with scyllo-inositol (100 mg/kg/day) or KMS88009 (10, 30 and 100 mg/kg/day) for 7 months from 5 to 12 months of age [11]. Behavioral tests were performed in the following sequence: Y-maze (day 1), Morris water maze (day 4 to 12) and contextual fear conditioning (day 26 to 27). To reduce stress, contextual fear conditioning was carried out 2 weeks after the Morris water maze task. In the therapeutic trial, APP/PS1 TG mice were treated with KMS88009 (30 mg/kg/day) for 3 months from 9 to 12 months of age. Y-maze tests short-term spatial working memory [12]. Y-maze is a 3-armed (40 L×10 W×12 H cm) horizontal maze in which the arms are symmetrically disposed at 120° angles from each other. Mice were placed at the end of one arm and allowed to move freely through the maze during a 5-min session. Arm entry was considered completed when the hind paws of the mouse were completely placed in the arm. Alternation was defined as successive entries into the 3 arms on overlapping triplet sets. The series of arm entries was recorded via video recorders. The percentage of alternation was defined according to the following equation: % alternation  =  [(number of alternations)/(total arm entries - 2)] ×100. Mice performing fewer than 7 alternations in 5 min were excluded from the subsequent analysis to accurately determine alternation scores. Morris water maze was used to measure spatial learning and memory [13]. A stainless pool apparatus with a diameter of 120 cm was used. A clear escape platform (circle, 12×12 cm) was located 1.0 cm below (hidden) the water surface containing white nontoxic paint. The temperature of water was kept constant throughout the experiment (21±1°C). The visual cues, for mice to recognize the environment and direction, are placed on the walls around the pool. The training was consisted of 8 consecutive days of testing with 4 trials per day. When a mouse was located on the escape platform, it was permitted to remain on the platform for 15 sec. If the mouse failed to find the platform within the maximum time (60 sec), it was placed on the platform for 15 sec. Then the subject was returned to its home cage and allowed to dry up after each trial. The platform location was kept constant and the starting position was varied. Each mouse was placed in the water with their nose pointing toward the wall at one of the starting points in a random manner. During each trial session, the time taken to find the hidden platform (latency) was recorded using a video camera-based Ethovision System (Nodulus, Nertherlands). On the 9^th^ day, each mouse was tested on a probe trial. The platform was removed from pool and the mice were allowed to swim for 60 sec to search. Swimming time was recorded during mice were in the pool quadrant where the platform had been previously located. Mice, which float or jump from platform, were eliminated from the experiment since these responses may be incompatible with the behaviors needed to locate and learn the platform position. In addition, animals with difficulty in swimming were also excluded from this study. Fear conditioning task was measured to test the ability to learn and remember an association between an aversive experience and environmental cues [14]. Contextual fear conditioning evaluation was performed using a fear-conditioning chamber (Courlbourn, USA) equipped with a computer-controlled fear conditioning system (FreActimetrics, USA). On the first day of training, each mouse was placed in the conditioning chamber and left to adjust for 2 min. Fear conditioning test was performed with conditional stimulus (CS) of sound (75 dB) for 20 sec followed by unconditional stimulus (US) of electric foot-shock (0.5 mA) for the last 2 sec in CS. After 1 min, the subject was returned to its home cage. After 24 hr, retention test was performed. The subject was placed in the same conditioning chamber and the behavior was observed for 5 min. Freezing response was measured without CS or US. Freezing behavior was defined as the complete absence of any movement except for respiration and heartbeat. Animals with no response to the foot-shock during training were excluded from the analysis. Data analyses including recordings of all behavioral responses were performed by keeping research colleagues blind.

### Quantification of Aβ in brain tissue

Mice subjected to behavior tests were sacrificed and levels of Aβ in their brains were analyzed. After removing olfactory lobe and cerebellum of brain, hippocampus and cerebral cortex were separated. The separated regions were Dounce-homogenized first in 10-fold mass of a lysis buffer (RIPA, 150 mM NaCl, 1% NP-40, 0.5% deoxycholic acid, 0.1% SDS and 50 mM tris, pH 8.0) or tris buffer (50 mM tris and pH 8.0) containing protease inhibitor cocktail (Pierce, USA) for prophylactic or therapeutic treatment groups, respectively. The homogenates were then centrifuged at 16,000 g for 20 min at 4°C. The supernatant (soluble Aβ fraction) was collected and stored at −70°C until analyzed. The resulting pellet was solubilized in 20-fold mass of cold guanidine solution (5 M guanidine HCl, 50 mM tris HCl and pH 8.0) at room temperature for 4 hr followed by dilution with cold Dulbecco's phosphate buffered saline (PBS) with 5% BSA and 0.03% tween-20. The diluent was centrifuged at 16,000 g for 20 min at 4°C to obtain the insoluble Aβ fraction. The tissue concentrations of Aβ40 and Aβ42 in the soluble and insoluble fraction were measured using Aβ-detecting sandwich enzyme-linked immunosorbent assay (ELISA) kit (BioSource, USA). The levels of soluble Aβ oligomers were measured by a dot-blot immunoassay with an anti-oligomer antibody and an anti-Aβ antibody on soluble fraction of hippocampus homogenates. The homogenate sample (3 µL) was spotted onto nitrocellulose membrane (Whatman, UK). After blocking with 5% BSA in tris-buffered saline (TBS) for 1 hr at room temperature, the membrane was incubated with rabbit anti-oligomer polyclonal antibody (A11; 1∶1,000; Millipore, USA) overnight at 4°C, followed by incubation with HRP-secondary anti-rabbit antibody (1∶5,000; Millipore, USA) for 1 hr at room temperature. Blots were developed using ECL reagents (PerkinElmer, USA). Blots were re-identified after oligomeric antibody was stripped and re-probing with the anti-Aβ antibody 6E10 (1∶1,000; Signet, UK). The density of dots was analyzed with Multi Gauge program (Fuji Film, Japan) and the data was normalized to the average density of the TG control group. Western blot analysis was carried out to analyze the reduced subtype of soluble Aβ oligomers. Soluble fraction of hippocampus homogenates were mixed with lithium dodecyl sulfate (LDS) sample buffer (Invitrogen, USA) and resolved by 4 to 12% Nu-PAGE gels (Invitrogen, USA) under non-reducing condition and transferred to a nitrocellulose membrane. Membrane was incubated in a 5% solution of non-fat dry milk for 1 hr at room temperature. After overnight incubation at 4°C with primary antibody (6E10, 1∶1,000), the membrane was incubated with HRP-secondary anti-mouse antibody (1∶2,000; Millipore, USA) for 1 hr at room temperature and developed using ECL reagents. Blots were stripped and re-probed with anti-β-actin antibody (1∶5,000; Sigma, USA) to confirm equal protein loading.

### Statistics

Data were expressed as the mean ± SEM or SD All data were analyzed for statistical significance by ANOVA followed with Tukey post-test or Dunnett's test. Value *P*<0.05 was taken as being statistically significant. Statistical analyses were performed using GraphPad Prism software.

## Results

### Physicochemical properties

Prior to *in vivo* examination, we assessed physicochemical properties of KMS88009. We found that pKa of KMS88009 was 1.5±0.12 in aqueous solution and log P of KMS88009 was 2.4±0.19 (Table S1 in File S1). As KMS88009 was soluble (583 µg/mL) in DMSO/tween-20/saline (Table S2 in File S1), DMSO/tween-20/saline was determined as a vehicle for KMS88009 to *in vivo* studies.

### Stability

To assess stability of KMS88009, we assessed microsomal and plasma stability tests in various species. We examined the rate of metabolism by measuring the disappearance of KMS88009 in LM of various species (Table 1). We found that the metabolic stability of KMS88009 in LM was the highest in humans and the lowest in dogs. The T_1/2_ values of KMS88009 were 35.2, 21.3, 58.4 and 65.3 min in monkeys, dogs, rats and mice, respectively. KMS88009 in human LM was stable until 60 min. The percentages of KMS88009 remaining in humans, monkeys, dogs, rats and mice were 92.7%, 30.9%, 14.8%, 61.2% and 52.4%, respectively. KMS88009 was stable in the plasma of humans, dogs, rats and mice for 6 hr at 37°C (Table S3 in File S1).

**Table 1:** Stability of KMS88009 in liver microsomes of various species.

Liver microsomes	T_1/2_ (min)		Remaining % of parent molecule at 60 min.	
	KMS88009	Positive Control	KMS88009	Positive Control
Human	√	12.3±0.9^a^	92.7±0.4	13.4±0.8^a^
Monkey	35.2±0.6	23.4±0.5^b^	30.9±0.2	16±0.4^b^
Dog	21.3±0.8	9.2±0.4^a^	14.8±0.2	4±0.2^a^
Rat	58.4±0.4	7.4±0.3^a^	61.2±0.2	ND^a^
Mouse	65.3±0.6	5.1±0.4^a^	52.4±0.3	ND^a^

Values are expressed as the mean ± SD of 3 independent experiments. T**_1/2_**: Half life, √: Mean percent value is confirmed over 90% at 60 min., ND: No-peaks detection, ^a^Verapamil as a positive control, ^b^Testosterone as a positive control.

### CYP inhibition

To assess the potential drug-drug interaction liabilities of KMS88009, we assess CYP inhibition assays. CYP inhibition affects plasma levels *in vivo* and induces adverse drug reactions or toxicity. The metabolic turnover of KMS88009 was assessed using five CYP isozymes (CYP3A4, 1A2, 2C9, 2C19, and 2D6) from human LM. We found that IC_50_ values of KMS88009 were 9.84 µM (CYP3A4), 0.74 µM (CYP1A2), 22.36 µM (CYP2C9), 2.51 µM (CYP2C19) and >50 µM (CYP2D6) (Table 2).

**Table 2:** Cytochrome P450 (CYP) enzyme inhibition assay of KMS88009.

Compound	IC_50_ (µM)				
	CYP3A4	CYP1A2	CYP2C9	CYP2C19	CYP2D6
Positive control	0.10^a^	4.45±0.21^b^	0.19±0.02^c^	3.69±0.25^d^	0.01^e^
KMS88009	9.84±0.15	0.74±0.12	22.36±1.25	2.51±0.21	>50

Values are expressed as the mean ± SD of 3 independent experiments. Positive control; ^a^Ketoconazole, ^b^Furafylline, ^c^Sulfaphenazole, ^d^Tranylcypromine, ^e^Quinidine.

### Pharmacokinetics in mice, rats, dogs and monkeys

**Figure 1:**
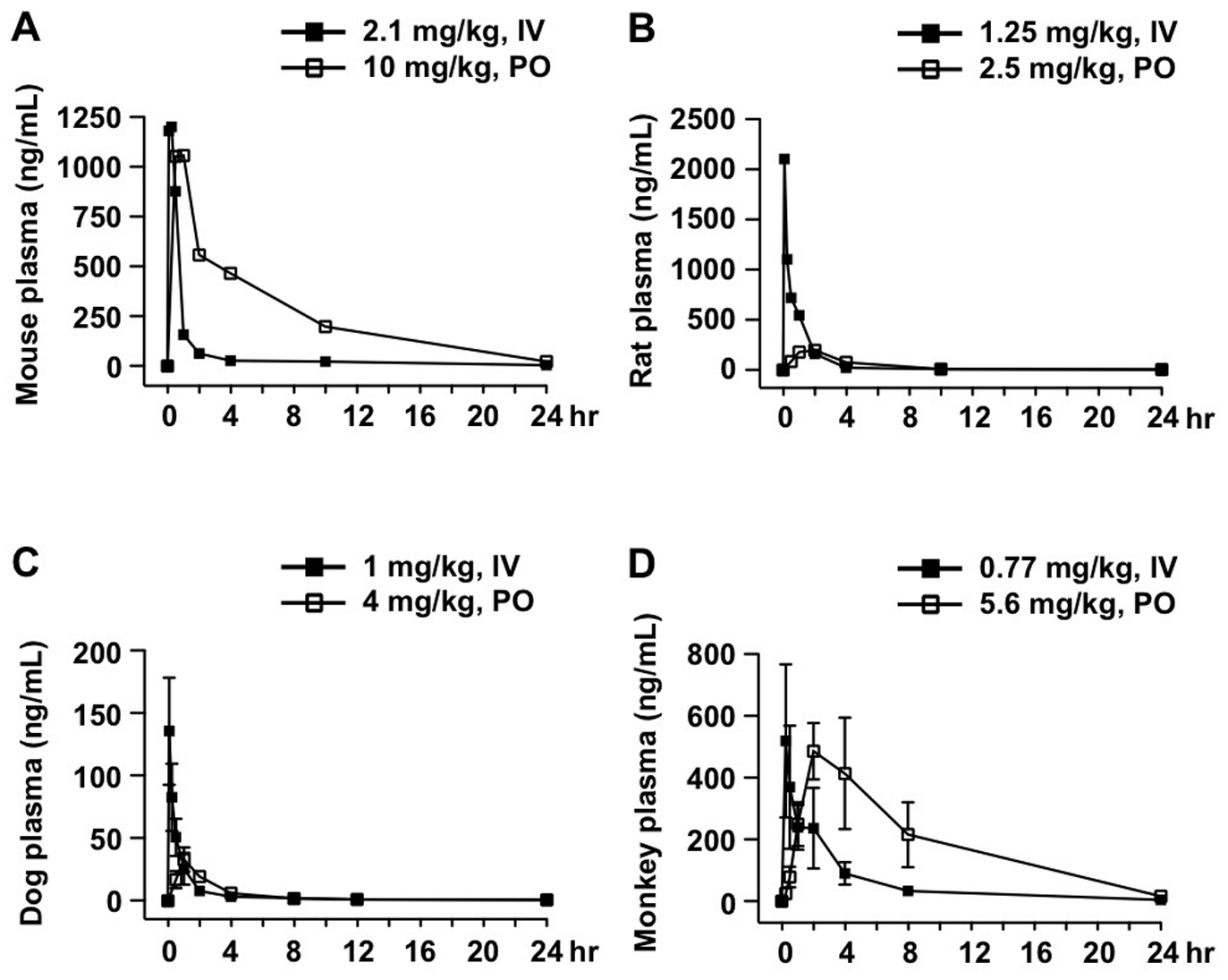
Pharmacokinetics of KMS88009. Plasma concentration versus time profiles of KMS88009 following IV or PO administration to (A) mice, (B) rats, (C) dogs and (D) monkeys. Plasma concentration of KMS88009 was determined after administration of single IV or PO dose. Each value was analyzed by LC-MS/MS and expressed as ng/mL. Data point of mice and rats represents the group average of 3 animals at each time-point and that of dogs and monkeys describes the mean ± SD of 3 animals.

**Figure 2:**
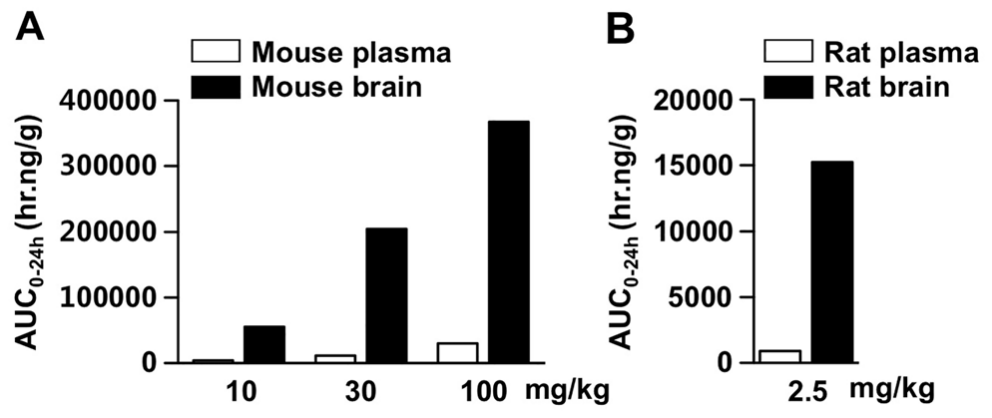
AUC_0–24h_ in brain and plasma after administration of KMS88009. AUC_0–24h_ of KMS88009 in plasma (white) or brain (black) was determined after oral administration to (A) mice and (B) rats. Each value represents the group average of 3 animals at each time-point.

Bioavailability and brain penetration properties are significantly important to drugs for brain disorders. Thus, we analyzed pharmacokinetic parameters and mean plasma concentration time curves for KMS88009 in various non-clinical species (Table 3 and Fig. 1). We found T_1/2_ and T_max_ as 4.0∼8.8 hr and 1.0∼2.7 hr, respectively and oral bioavailability of KMS88009 as 99.6, 27.8, 21.6 and 34.1% in mice, rats, dogs and monkeys, respectively. We observed high penetration of KMS88009 across the blood-brain barrier (BBB) in mice and rats (Figure 2). Level of KMS88009 (AUC_0–24h_) in the brain was over 12-fold higher than that in the plasma in mice (Table 4). In rats, concentration of KMS88009 in the brain was 16.9-fold higher than that in the plasma (Table 4). In addition, we analyzed dose proportionality of KMS88009 in mice from 10 to 100 mg/kg (Table 4). We found that the ratios of AUC_0–24h_ and C_max_ of KMS88009 (10, 30 and 100 mg/kg) were 1.0∶2.9∶6.7 and 1.0∶2.2∶3.7, in the plasma, respectively, and 1.0∶3.7∶6.6 and 1.0∶2.8∶3.7, in the brain, respectively. Therefore, We observed dose-dependent absorption of KMS88009 from 10 to 100 mg/kg without linear relationship between AUC_0–24h_ and C_max_.

**Table 3:** Pharmacokinetic parameters of KMS88009 following IV or PO administration to mice, rats, dogs, and monkeys.

Species	Routes	Dose (mg/kg)	Pharmacokinetic parameters								
			AUC_0–24h_ (hr.ng/ml)	AUC_INF_ (hr.ng/ml)	C_max_ (ng/ml)	T_max_ (hr)	T_1/2_ (hr)	Vd (L/kg)	Cl (L/hr*kg)	MRT_last_ (hr)	BA (%)
Mouse	IV	2.1	1298.1	1329.7	-^a^	-	5.9	32.1	3.8	2.9	
	PO	10	6156.2	6309.4	1058.2	1.0	4.6	-^a^	-	5.5	99.6
Rat	IV	1.25	1637.5	1672.3	-	-	7.2	8.0	0.8	1.8	
	PO	2.5	908.5	928.8	215.0	1.7	4.0	-^a^	-	4.0	27.8
Dog	IV	1	112.2±43.4	117.0±43.6	-	-	8.1±1.4	114.8±61.1	9.5±3.9	2.9±0.6	
	PO	4	97.1±14.0	103.1±13.0	32.8±9.8	1.0±0.0	8.8±2.5	-^a^	-	3.8±0.4	21.6
Monkey	IV	0.77	1539.8±550.0	1585.2±576.5	-	-	5.8±0.8	4.4±1.1	0.5±0.2	3.6±0.5	
	PO	5.6	3814.3±1430.0	3917.2±1478.2	504.6±78.3	2.7±1.2	4.3±0.2	-	-	5.9±0.6	34.1

Values of mice and rats are expressed the group average of 3 animals at each time-point and dogs and monkeys are expressed as the mean ± SD of 3 animals. ^a^Not calculated.

**Table 4:** Pharmacokinetic parameters of KMS88009 in plasma and brain after PO administration to mice and rats.

	Pharmacokinetic parameters	Mice			Rats
		10 mg/kg	30 mg/kg	100 mg/kg	2.5 mg/kg
Plasma	AUC_0–24h_ (hr.ng/g)	4565.6	13381.6	30532.4	908.5^a^
	C_max_ (ng/g)	743.6	1638.8	2714.7	215.0^a^
	T_max_ (hr)	1.0	1.0	2.0	1.7^a^
Brain	AUC_0–24h_ (hr.ng/g)	55395.5	205268.3	367538.5	15241.9
	C_max_ (ng/g)	6368.0	17813.0	23314.0	1309.0
	T_max_ (hr)	2.0	4.0	10.0	2.0

Values are expressed as the group average of 3 independent animals. ^a^Values are the same with the data of rats in table 3.

### Toxicity

Many therapeutically effective drug candidates fail in clinical trials due to their toxicity. To examine safety of KMS88009, we performed hERG assay and single/repeat dose toxicity study. We found that KMS88009 weakly inhibited the hERG potassium channel (IC_50_  = 52.14 µM) (Table S4 in File S1) and was well tolerated with no side effects after single and 2-week repeated administration in rats (Table S5–11 in File S1). A single oral dose of KMS88009 produced no toxic effects in SD rats at the dose of 2,000 mg/kg, and the median lethal dose (LD_50_) was estimated to be greater than 2,000 mg/kg (Table S5 and S6 in File S1). 2-Week repeated oral administration of KMS88009 did not cause any toxic effect to SD rats at the dose level of 1,000 mg/kg/day and, thus, no-observed-adverse-effect-level was considered to be over 1,000 mg/kg. (Table S5, S7–11 in File S1). In both single and repeated administration toxicity assessment, we did not observe any mortality, symptomatic signs or body weight changes in all subjected animals (Table S5–8). We found all hematological and serum alternations remained within normal limits except for reticulocyte and T-Bil, respectively (Table S9 and S10) in repeated administration study. We observed significant increase of reticulocyte and decrease of T-Bil in the 100 mg/kg/day group compared to the vehicle-treated group. Necropsy did not reveal any gross pathological changes. In addition, we measured mean weight of organs and organ-to-body ratio of rats in the repeated administration toxicity study. (Table S11). We observed no significant alternation of organ weights, except for the decreased brain weight of the 300 mg/kg/day group. However, the substantial differences of T-Bil (100 mg/kg/day) and brain weight (300 mg/kg/day) were not dose-dependent.

Safety and BBB penetration property is of crucial features for AD drug candidates, as treatments usually require long-term oral administration. Collectively, our ADME/Tox assessment indicates that orally administered KMS88009 is stable, safe and significantly crossing BBB.

### Prophylactic efficacy against AD-like behaviors

**Figure 3:**
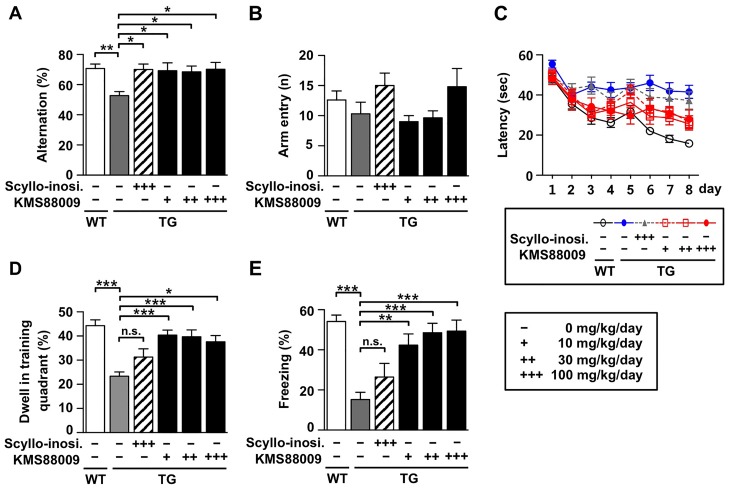
Recovered memory deficits by KMS88009 in APP/PS1 transgenic mice. Scyllo-inositol or KMS88009 were orally administered to 5-month-old transgenic (TG) mice (before symptomatic signs of disease) for 7 months. Age-matched wild type (WT) mice were used as controls. Spontaneous alternation and contextual fear conditioning tests were performed. (A) Spontaneous alternation behavior and (B) numbers of arm entries during 5-min session were measured. (C) Training trials and (D) probe trials during 1-min session were measured (Table S12). (E) Contextual fear conditioning was measured in identical conditioning chamber during 5-min session at 24 hr after training. Scyllo-inosi. in each figure means scyllo-inositol. WT, n = 18∼30; TG, n = 13∼16; scyllo-inositol 100 mg/kg/day, n = 9∼13; KMS88009 10 mg/kg/day, n = 10∼17; KMS88009 30 mg/kg/day, n = 13∼18; KMS88009 100 mg/kg/day, n = 9∼14. The data represents the mean ± SEM, *P<0.05, **P<0.01, ***P<0.001, **n.s**: no significance.

To assess the prophylactic efficacy of KMS88009 against development of AD-like phenotypes, we treated APP/PS1 TG mice (male) before the onset of learning and memory deficits. We orally administered KMS88009 to TG mice from 5 months of age (∼4 weeks before symptom onset) until 12 months of age for 7 months. Scyllo-inositol, currently in clinical trials for the therapeutic and preventive effects on AD by targeting Aβ, was used as a control compound [11]. In the Y-maze test, we observed the frequency of spontaneous alternation of APP/PS1 TG mice (vehicle-treated, 12-month-old) was substantially lower than that of age-matched WT mice (*P*<0.01, Fig. 3A). Oral administration of scyllo-inositol (100 mg/kg/day: 70.0%) or KMS88009 (10 mg/kg/day: 69.3%; 30 mg/kg/day: 68.5%; 100 mg/kg/day: 70.2%) (*P*<0.05) reversed the reduced frequency of spontaneous alternation of 12-month-old APP/PS1 TG mice. The number of arm entries did not differ significantly among the experiment groups, indicating that general locomotor activity was not affected by chronic scyllo-inositol or KMS88009 treatment (Fig. 3B). Consistent with Y-maze tests, vehicle-treated APP/PS1 TG mice exhibited longer escape latencies throughout the training trials compared to the performance of the age-matched WT mice. On the contrary, we observed significant decrease of the time to reach the hidden platform (latency) during the 8-day training trial period by KMS88009, indicating active spatial learning ability (Fig. 3C, Table S12). KMS88009-treated APP/PS1 TG mice showed significantly shorter escape latencies starting on the 6^th^ day of the training trials. The strength of the learned spatial search bias was assessed during a probe trail on the 9th day without the hidden platform (Fig. 3D). The time % for each mouse was defined as the time spent in the target quadrant of the original platform location after removal of the hidden platform. Mice in all groups, except for the vehicle-treated TG group, spent more time in the target quadrant than would be expected for free swimming. The swimming time % within the target quadrant was significantly lower for the vehicle-treated APP/PS1 TG mice (23.4%) compared to the WT controls (44.3%) (*P*<0.001). However, the reduced swimming time % within the target quadrant for TG mice was significantly increased by KMS88009 treatment by 40.4% (10 mg/kg/day, *P*<0.001), 39.7% (30 mg/kg/day, *P*<0.001) and 37.6% (100 mg/kg/day, *P*<0.05). In the contextual fear conditioning test, the total percentage of time spent freezing during subsequent re-exposure to the same training chamber significantly decreased for the vehicle-treated TG mice (15.3%) compared to the WT controls (54.1%) (*P*<0.001, Fig. 3E). We observed significant increase of the freezing responses (%) of APP/PS1 TG mice by prophylactic treatment of KMS88009 by 42.3% (10 mg/kg/day, *P*<0.01), 48.6% (30 mg/kg/day, *P*<0.001) and 49.3% (100 mg/kg/day, *P*<0.001). Overall, KMS88009 significantly prevented AD-like symptom onset in APP/PS1 TG mice compared with the mice treated with scyllo-inositol, which is prophylactically effective, and even their WT littermates.

### Prophylactic efficacy against Aβ accumulation

**Figure 4:**
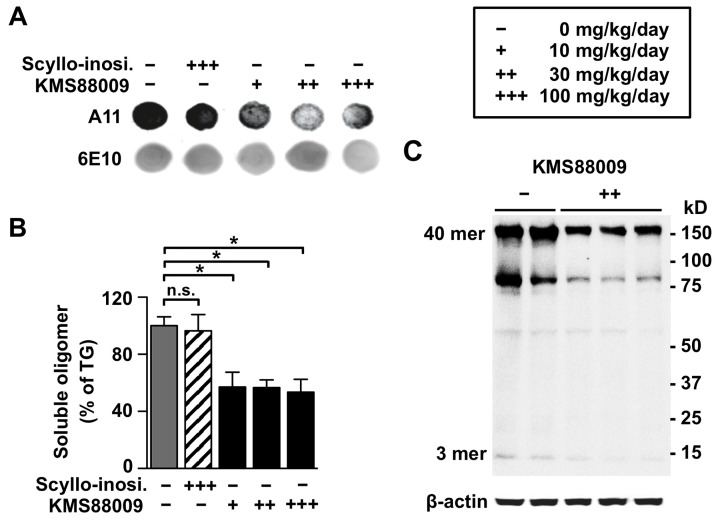
Reduced hippocampal oligomeric Aβ by KMS88009 in APP/PS1 TG mice. Scyllo-inositol or KMS88009 were orally administered to 5-month-old transgenic (TG) mice (before symptomatic signs of disease) for 8 months. Each hippocampus from TG mice was sequentially extracted with RIPA (soluble fraction) and guanidine (insoluble fraction) solution. (A) Dot blots and (B) densitometry of blots for oligomers (A11) and Aβ (6E10). (C) Western blots for Aβ oligomers in hippocampus homogenates. Scyllo-inosi. in each figure means scyllo-inositol. TG, n = 16; scyllo-inositol 100 mg/kg/day, n = 13; KMS88009 10 mg/kg/day, n = 17; KMS88009 30 mg/kg/day, n = 18; KMS88009 100 mg/kg/day, n = 14. The data represents the mean ± SEM, *P<0.05, **P<0.01, ***P<0.001, **n.s**: no significance.

To examine preventive effects of KMS88009 against Aβ accumulation in AD brains, the mice were sacrificed after behavioral tests and their brains were examined. We measured alternation of soluble Aβ oligomer levels by a dot-blot immunoassay with A11 (anti-oligomer) and 6E10 (anti-Aβ) antibodies in the soluble fraction of hippocampus (Fig. 4A). KMS88009-treated TG groups significantly reduced levels of soluble oligomeric Aβ by 43.1% (10 mg/kg/day), 43.2% (30 mg/kg/day) and 46.4% (100 mg/kg/day), respectively, compared to the Aβ levels in the vehicle-treated TG group (Fig. 4B, *P*<0.05). There was no statistically significant difference in the soluble oligomeric Aβ level between the KMS88009-treated groups. To determine which species of Aβ oligomers were principally affected in the brains of KMS88009-treated mice, we performed western blot assays with the soluble fractions of hippocampal homogenates (Fig. 4C). We found that KMS88009 prevented the accumulation of low-molecular-weight (3-mer) and high-molecular-weight (55 to 180 kDa) Aβ oligomeric species without effecting insoluble species. KMS88009-treated TG groups showed significantly reduced soluble Aβ42 levels in the hippocampus and cortex, by 47∼49% and 59∼66%, respectively, compared to the vehicle-treated TG mice (Table 5, *P*<0.01). KMS88009 treatment also resulted in significant reductions in the soluble Aβ40 level except in the hippocampus of the 10 mg/kg/day-treated group; the reductions were 34∼36% in the hippocampus and 44∼60% in the cortex (*P*<0.01 compared with the TG group) (Table 5). Only the administration of KMS88009 at 100 mg/kg/day was able to reduce the level of insoluble Aβ40 in the hippocampus of TG mice (*P*<0.05, Table 5). As antibody 6E10 also can detect amyloid precursor protein (APP), it is possible that secreted APP was visualized on western-blot and found decreased by KMS88009. Although the APP reduction hypothesis is another considerable feature of KMS88009, it requires in-depth study to investigate.

**Table 5:** Aβs level in TG mice brain after KMS88009 (10, 30 and 100 mg/kg/day) administration for 8 months.

	Hippocampus				Cortex			
Group	Aβ40 (ng/g wet brain)		Aβ42 (ng/g wet brain)		Aβ40 (ng/g wet brain)		Aβ42 (ng/g wet brain)	
	Soluble^a^	Insoluble	Soluble^a^	Insoluble	Soluble^a^	Insoluble	Soluble^a^	Insoluble
Vehicle	1.94±0.15	2048.0±240.3	27.49±2.61	4105.0±327.4	1.83±0.18	3938.0±372.0	48.09±4.20	3335.6±1147.6
Scyllo-inositol (100 mg/kg)	1.81±0.20	2053.0±415.8	26.17±5.88	3834.0±603.2	1.99±0.18	4073.0±345.7	50.04±4.41	3525.0±1242.1
KMS88009 (10 mg/kg)	1.53±0.14	1691.0±278.9	13.48±2.67** †	3725.0±466.1	1.01±0.09^***†††^	3038.0±231.8	17.84±2.71^***†††^	3178.9±1132.4
KMS88009 (30 mg/kg)	1.23±0.10** †	1485.0±265.6	13.37±2.43** †	4088.0±371.7	0.92±0.10^***†††^	3784.0±402.2	19.66±2.68^***†††^	3129.3±1011.4
KMS88009 (100 mg/kg)	1.27±0.17** †	1062.0±198.3†	12.92±2.57** †	3771.0±341.7	0.74±0.06^***†††^	4366.0±362.9	16.19±1.74^***†††^	4038.3±1385.9

The data represents the mean ± SEM.

a RIPA-buffer soluble fraction.

**P<0.01, ***P<0.001 vs vehicle control group.

† *P*<0.05, ^†††^ *P*<0.001 vs the Scyllo-inositol group.

### Therapeutic efficacy against AD-like behaviors

**Figure 5:**
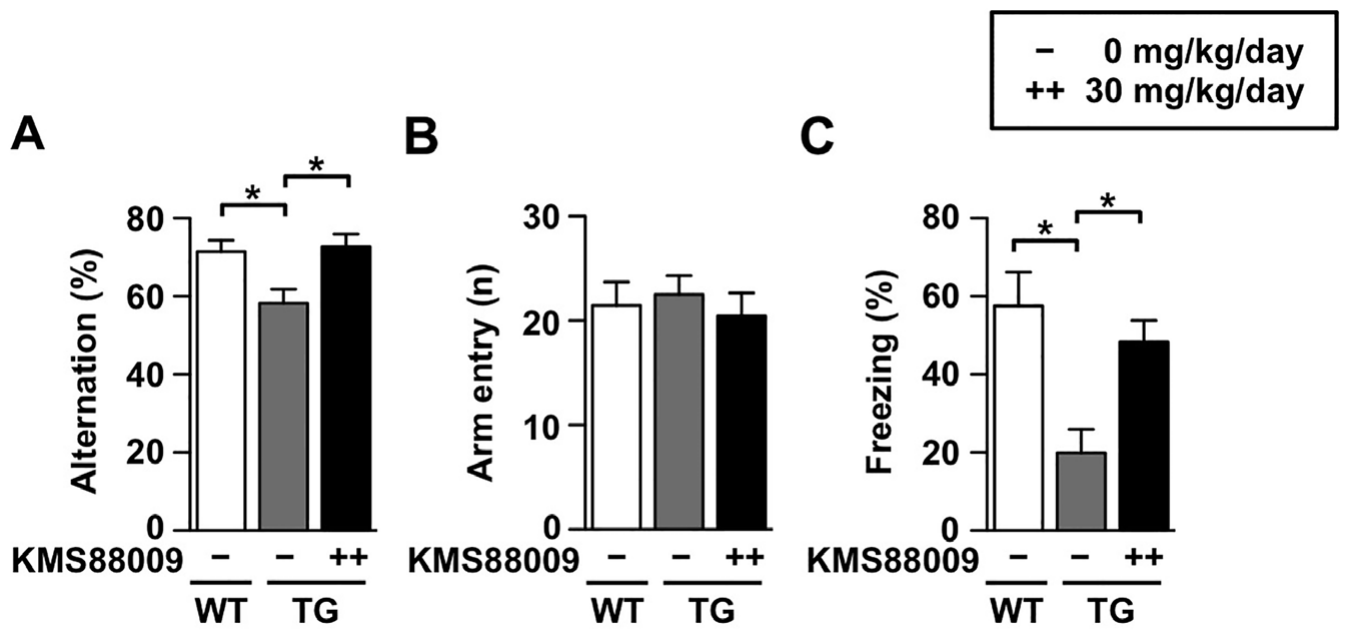
Improved memory deficits by KMS88009 in APP/PS1 transgenic mice. Scyllo-inositol or KMS88009 (30 mg/kg/day) were orally administered to 9-month-old transgenic (TG) mice (with symptomatic signs of disease) for 3 months. Age-matched wild type (WT) mice were used as controls. Spontaneous alternation was performed on behavioral testing day 1. (A) Spontaneous alternation behavior and (B) the numbers of arm entries during 5-min session were measured. (C) Contextual fear conditioning was measured in identical conditioning chamber during 5-min session at 24 hr after training. WT, n = 11; TG, n = 11; KMS88009 30 mg/kg/day, n = 11. The data represents the mean ± SEM, *P<0.05.

To assess the therapeutic efficacy of KMS88009 against already-developed AD-like behaviors in aged AD mice, we orally administered KMS88009 to APP/PS1 TG mice after the onset of AD-like phenotypes. KMS88009 (30 mg/kg/day) was orally administered to mice from 9 months of age until 12 months of age. Spontaneous alternation of APP/PS1 TG mice was significantly reduced by 3-month KMS88009 treatment (Fig. 5A, *P*<0.05). The number of arm entries was not significantly different among the tested groups (Fig. 5B). In addition, the reduced freezing response (20.0%) in APP/PS1 TG mice was significantly reversed by treatment with KMS88009 (48.3%) (Fig. 5C, *P*<0.05). The abnormal cognitive behaviors of APP/PS1 TG mice were recovered to the WT level after 3 months of daily administration.

### Therapeutic efficacy against Aβ accumulation

To examine therapeutic effects of KMS88009 against Aβ accumulation in the brains of aged AD mice, the concentrations of Aβ40 and Aβ42 in the hippocampus were measured. We observed that, in the hippocampus of APP/PS1 TG mice, KMS88009 decreased both the soluble (19.4%) and insoluble Aβ40 levels (44.9%), compared to those in TG mice. However, this difference was not statistically significant (Table 6). On the contrary, KMS88009 treatment significantly decreased soluble Aβ42 level (44.3%) compared to vehicle-treated TG controls (*P*<0.01). KMS88009 also significantly decreased the insoluble Aβ42 level, by 50.9%, compared to the level in TG littermates (*P*<0.001).

**Table 6:** Aβs level in hippocampus of TG mice after KMS88009 (30 mg/kg/day) administration for 3 months.

Group	Aβ40 (ng/g wet brain)		Aβ42 (ng/g wet brain)	
	Soluble^a^	Insoluble	Soluble^a^	Insoluble
Vehicle	0.38±0.05	383.8±104.3	2.09±0.26	2855.0±327.8
KMS88009 (30 mg/kg)	0.31±0.04	211.4±37.1	0.91±0.13**	1401.0±188.4^***^

The data represents the mean ± SEM.

a Tris-buffer soluble fraction.

**P<0.01, ***P<0.001 vs the TG control group.

Taken together, prophylactic and therapeutic treatment of KMS88009 reduced AD-like behaviors and Aβ deposition in APP/PS1 TG mice. When orally administered for 7 months before the onset of AD-like phenotypes, KMS88009 prevented cognitive deficits and oligomeric Aβ deposition. KMS88009 also rescued short-term spatial working memory and decreased levels of soluble and insoluble of Aβ42 when therapeutically treated to aged APP/PS1 mice after the development of AD-like phenotypes.

## Discussion

In the current study, we report stability, toxicity and pharmacokinetics profiles of KMS88009 in various species including mice, rats, dogs, monkeys and humans, followed by learning and memory behavior tests in APP/PS1 Alzheimer's mice. Orally administered KMS88009 rescues behavioral dysfunctions, by reducing Aβ oligomer formation in the APP/PS1 mice models, prophylactically and therapeutically.

Although the amyloid cascade hypothesis has been investigated as one of major causes of AD, drug candidates targeting the production and aggregation of Aβ have not been approved for the clinical use to date [1], [2], [15]. Such disappointments in AD clinical trials support the view that the Aβ abnormality begins long before the onset of cognitive loss and that prophylactic strategy is critical in AD [6], [16], [17]. However, therapeutic approach is also necessary for AD treatment as current clinical diagnosis lack ability to sort at-risk AD individuals before the development of cognitive loss. Thus, preventively and therapeutically effective mode of action against Aβ abnormality and cognitive deficits can be an attractive feature for AD drug candidates. Therefore, KMS88009 holds potential to be a promising drug candidate for the treatment of AD at various stages based on its ability to reduce the levels of Aβ oligomers, leading to substantial improvements in abnormal AD behaviors.

Interestingly, in this study, KMS88009 induced partial reduction of Aβ aggregates. Prophylactic treatment of KMS88009 decreased levels of soluble Aβ40 and Aβ42 while therapeutic approach reduced those of soluble and insoluble Aβ42 only. However, in both manners, KMS88009 substantially attenuated hippocampus-dependent behavioral deficits. The results of the present study suggest that KMS88009 exerts its beneficial effects through the reduction of both low- and high-molecular-weight oligomeric Aβ42 species and indirectly speak to considerable debate as to which species of Aβ are the most neurotoxic [18].

## Supporting Information

File S1 Methods for measurements of pKa and log P, solubility studies, plasma stability studies and hERG inhibition assay are available. Tables S1-12 are also available.
(DOCX)
