# Supplementary material for: Aminostyrylbenzofuran Directly Reduces Oligomeric Amyloid-β and Reverses Cognitive Deficits in Alzheimer Transgenic Mice
Source: PLoS One. 2014 Apr 23;9(4):e95733. doi: 10.1371/journal.pone.0095733 (PMC3997483; doi:10.1371/journal.pone.0095733)
Supplement: File S1 — Methods for measurements of pKa and log P, solubility studies, plasma stability studies and hERG inhibition assay are available. Tables S1-12 are also available. (DOCX) [file pone.0095733.s001.docx]

**Aminostyrylbenzofuran directly reduces oligomeric amyloid-β and reverses cognitive deficits in Alzheimer transgenic mice**

Sang-Hyun Lee^†,1,2^, YoungSoo Kim^†,3,4^, Hye Yun Kim^3,4,5^, Young Hoon Kim^1^, Maeng Sup Kim^1^, Jae Yang Kong^6^, Mun-Han Lee^2^, Dong Jin Kim^3,*^ and Young Gil Ahn^1,*^

^1^Hanmi Research Center, Hanmi Pharmaceutical Co., Ltd., Hwaseong-si, Gyonggi-do, Republic of Korea, ^2^Department of Biochemistry, College of Veterinary Medicine, Seoul National University, Seoul, Republic of Korea, ^3^Center for Neuro-Medicine, Brain Science Institute, Korea Institute of Science and Technology, Seoul, Republic of Korea, ^4^Biological Chemistry Program, University of Science and Technology (UST), Daejeon, Republic of Korea, ^5^Department of Biochemistry and Biomedical Sciences, Seoul National University, College of Medicine, Seoul, Republic of Korea and ^6^College of Pharmacy, Keimyung University, Daegu, Republic of Korea

^†^These authors contributed equally.

Corresponding authors:

Dong Jin Kim; djk2991@kist.re.kr; Tel.: +82-2-958-5142; Fax: +82-2-958-5189

Young Gil Ahn; ygahn@hanmi.co.kr; Tel.: +82-31-371-5071; Fax: +82-31-371-5119

**Methods**

**Dissociation constant (pKa) and partition coefficient (log *P*)** Dissociation constant (pKa) and partition coefficient (log *P*) of KMS88009 were measured by a pH-method using GLpKa (Sirius, UK). pKa was measured by titrating KMS88009 solution and calculated from the shape of the titration curve. We dissolved 10 mg KMS88009 in 150 mM NaCl in a GLpKa beaker, adjusted the solution to pH 2.0 with 0.5 N HCl and titrated up to pH 10.0 with 0.5 N KOH for pKa calculation. Log *P* of KMS88009 was calculated by a two-phase acid-base titration in a mixture of water and octanol. Three separate potentiometric titrations of KMS88009 were carried out at 23 ℃ in various octanol-water (0.15 M KCl) mixtures (0.5% to 50% octanol). The initial pH was adjusted to 10.0 with standardized 0.5 M NaOH prior to each titration. Titrations were performed with standardized 0.5 M HCl to pH 1.8. The pKa and log *P* values were calculated using RefinementPro software (Sirius, UK). Values are expressed as the mean ± SD of 3 independent experiments.

**Solubility studies** Solubility of KMS88009 in distilled water (DW), simulated gastric fluid (SGF, pH 1.2), simulated intestinal fluid (SIF, pH 6.8) and DMSO/tween-20/saline was determined. Excess amounts of KMS88009 were placed in screw-capped vials containing 2 mL of DW, SGF, SIF and DMSO/tween-20/saline. Suspensions were vortexed for 2 min and kept in a shaking water-bath at 37 °C. We transferred 250 μL of each sample to the filter insert of centrifuge tubes. After centrifugation at 16,000 rpm for 15 min, the filtrates of DW, SGF and SIF were analyzed by HPLC (Agilent 1200 series, USA). Values are expressed as the mean ± SD of 2 independent experiments.

**Plasma stability studies** Plasma stability of the KMS88009 was studied in human, dog, rat and mouse plasma. Each plasma from different species was diluted to 5-fold with 0.05 M phosphate buffered saline (PBS, pH 7.4 at 37 °C). We added KMS88009 to 1 mL of preheated plasma solution to yield a final concentration of 1 μM. The assay was performed in a shaking water bath at 37 °C and conducted in triplicate. Samples (50 μL) were taken at 0, 0.5, 1, 2 and 6 hr and added to 10 μL of 0.05% formic acid. The samples were subjected to vortex mixing for 1 min and centrifuged at 4 °C for 20 min at 14,000 rpm. The clear supernatants were analyzed by liquid chromatography/mass/mass spectrometry (LC-MS/MS). Values are expressed as the mean ± SD of 2 independent experiments. Stability is expressed as the percentage of remaining compound in % relative to the start concentration set to 100%.

**Inhibitory effect on hERG potassium ion channel** A hERG-expressed HEK-hERG cell line (IonGate Biosciences, Frankfurt, Germany Co.) was cultured in a DMEM (Dulbecco's Modified Eagle's Medium, Sigma Co., St. Louis, MO, USA) including 10% fetal bovine serum (FBS, Cambrex, Walkersville, MD, USA) and 0.5 mg/mL zeocin (Invitrogen, Carlsbad, CA, USA) to 80% confluency. To measure the potassium ion current, the solution comprising 115 mM K-aspartate, 20 mM KCl, 10 mM EGTA, 10 mM HEPES, 2.5 mM tris-phosphocreatine, 0.1 mM Na_2_GTP and 5 mM MgCl_2_ (pH 7.2) was used as an electrode inside solution, and the solution comprising 135 mM NaCl, 5 mM KCl, 1 mM MgCl_2_, 2 mM CaCl_2_, 10 mM glucose and 10 mM HEPES (pH 7.2) was used as an extracellular perfusate. Drug was prepared by diluting with extracellular perfusate to be a desired concentration. The prepared test drug was placed in a 7-array polyethylene tube connected into a capillary for gas chromatography and was dropped at 100 μM or less of height to be added into HEK-hERG cell line by gravity. Potassium current was measured using an EPC10 (Instrutech Co., NY, USA) patch clamp amplifier in accordance with the conventional whole-cell patch clamp method. Electrode used in the measurement was prepared from a borosilicate glass capillary (external diameter: 1.65 mm, inside diameter: 1.2 mm, Corning 7052, Garner Glass Co., Claremont, CA, USA) using P-97 Flaming-Brown micropipette puller (Sutter Instrument Co.). The electrode was coated with Sylgard 184 (Dow Corning Co., Midland, MI, USA) and trimmed with microforge (Narishige Co., Tokyo, Japan). The culture dish including cell was placed in the inverted microscope (Nikon Co.) and extracellular fluid was perfused at 1 ~ 2 mL/min of perfusion rate. Over 80% of membrane capacitance and series resistance of the cell membrane was calibrated and ion current was measured at 2 kHz of sampling rate and 2 kHz (-3 dB; 8-pole Bassel filter) of low-pass filter. The test was conducted at room temperature (21 ~ 24 ℃). The data was analyzed using Pulse/Pulsefit (v9.0, HEKA Elcktronik, Lambrecht, Germany) and Igor macro. The results are given as mean ± SEM. A concentration-response curve was adjusted by Hill equation [Block = (1+IC_50_/[drug]n)-1] to enumerate IC_50_, the concentration at which 50% ion current was observed.

**Table S1. Dissociation constant (pKa) and partition coefficient (log *P*) of KMS88009.**

| Parameter | Value |
| --- | --- |
| pKa | 1.5 ± 0.12 |
| log *P* | 2.4 ± 0.19 |

Values are expressed as the mean ± SD of three independent experiments.

**Table S2. Solubility of KMS88009**

| Solution | Solubility (mg/kg) |
| --- | --- |
| DW | 0.000 |
| SGF | 0.004 |
| SIF | 0.000 |
| DMSO/tween-20/saline | 0.583 |

Distilled water (DW), simulated gastric fluid (SGF, pH 1.2), simulated intestinal fluid (SIF, pH 6.8) and DMSO/tween-20/saline (3.3:19.8:79.6).

Values are expressed as means of two independent experiments.

**Table S3. Stability of KMS88009 in human, dog, rat and mouse plasma.**

| Plasma | Parent compound remaining (%) | | | | |
| --- | --- | --- | --- | --- | --- |
|  | Incubation time (hr) | | | | |
|  | 0 | 0.5 | 1 | 2 | 6 |
| Human | 100.0 | 100.0 | 100.0 | 82.0 | 100.0 |
| Dog | 100.0 | 100.0 | 100.0 | 100.0 | 100.0 |
| Rat | 100.0 | 86.9 | 93.7 | 100.0 | 100.0 |
| Mouse | 100.0 | 100.0 | 100.0 | 100.0 | 100.0 |
| PBS buffer | 100.0 | 99.1 | 88.6 | 100.0 | 100.0 |

Values are expressed as means of two independent experiments.

**Table S4. Inhibitory efficacy of KMS88009 for hERG channel currents in voltage-clamped HEK293 cells.**

| Compound | KMS88009 |
| --- | --- |
| IC_50_ (μM) | 52.14 ± 3.68 μM |

Values are expressed as the mean ± SEM of three independent experiments.

**Table S5. Mortality and symptomatic signs after single or repeated (2-week) administration of KMS88009 in rats.**

| Dose (mg/kg/day) | Single administration | | Repeated administration | | |
| --- | --- | --- | --- | --- | --- |
|  | 1,000 | 2,000 | 100 | 300 | 1,000 |
| Mortality | 0 / 3 ^a^ | 0 / 3 | 0 / 5 | 0 / 5 | 0 / 5 |
| Symptomatic signs | - ^b^ | - | - | - | - |

^a^Values are expressed as numbers of dead animals / total number of animals. ^b^No clinical sings were observed.

**Table S6. Body weight after single administration of KMS88009 in rats.**

| Days | Dose (mg/kg/day) | | |
| --- | --- | --- | --- |
|  | 0 ^a^ | 1,000 | 2,000 |
| 1 | 163.0 ± 4.6 | 164.4 ± 4.2 | 162.2 ± 3.6 |
| 4 | 197.7 ± 7.6 | 195.2 ± 7.6 | 185.4 ± 6.3 |
| 8 | 206.8 ± 3.6 | 207.5 ± 13.0 | 200.2 ± 7.4 |
| 11 | 229.0 ± 3.8 | 228.1 ± 11.1 | 220.2 ± 10.0 |
| 14 | 235.9 ± 7.3 | 233.1 ± 16.6 | 228.6 ± 12.2 |

Body weight (g). Values are expressed as the mean ± SD. ^a^Vehicle control group.

**Table S7. Abnormal gross findings of necropsy after single or repeated (2-week) administration of KMS88009 in rats.**

| Dose  (mg/kg/day) | Single administration | | Repeated administration | | | |
| --- | --- | --- | --- | --- | --- | --- |
|  | 1,000 | 2,000 | | 100 | 300 | 1,000 |
| Abnormal gross findings | 0 / 3 | 0 / 3 | | 0 / 5 | 0 / 5 | 0 / 5 |

Values are expressed as numbers of macroscopic changed animals / total number of animals.

**Table S8. Body weight after repeated (2-week) administration of KMS88009 in rats.**

| Days | Dose (mg/kg/day) | | | |
| --- | --- | --- | --- | --- |
|  | 0 ^a^ | 100 | 300 | 1,000 |
| 1 | 228.0 ± 8.5 | 227.6 ± 7.8 | 227.8 ± 7.1 | 228.3 ± 6.5 |
| 4 | 250.8 ± 9.6 | 253.1 ± 7.0 | 250.5 ± 9.9 | 251.1 ± 8.6 |
| 8 | 292.7 ± 13.5 | 297.2 ± 9.3 | 294.2 ± 14.6 | 296.4 ± 14.2 |
| 11 | 318.7 ± 17.2 | 321.2 ± 13.1 | 317.6 ± 17.0 | 323.0 ± 15.8 |
| 14 | 340.7 ± 18.8 | 344.4 ± 17.1 | 338.5 ± 16.9 | 346.6 ± 16.1 |

Body weight (g). Values are presented as the mean ± SD. ^a^Vehicle control group.

**Table S9. Hematological changes after repeated (2-week) administration of KMS88009 in rats.**

| Parameters | Dose (mg/kg/day) | | | | |
| --- | --- | --- | --- | --- | --- |
|  | 0 ^a^ | 100 | 300 | 1,000 | |
| RBC (x10^6^/μL) | 6.90 ± 0.25 | 7.25 ± 0.46 | 6.93 ± 0.26 | 7.14 ± 0.27 | |
| Hemoglobin (g/dL) | 13.7 ± 0.5 | 14.3 ± 1.0 | 13.9 ± 0.7 | 14.0 ± 0.8 | |
| Hematocrit (%) | 41.0 ± 1.7 | 43.2 ± 2.6 | 41.4 ± 1.9 | 42.1 ± 2.2 | |
| MCV (fL) | 59.4 ± 1.0 | 59.7 ± 1.3 | 59.7 ± 1.5 | 59.0 ± 1.0 | |
| MCH (pg) | 19.9 ± 0.4 | 19.8 ± 0.5 | 20.0 ± 0.6 | 19.6 ± 0.5 | |
| MCHC (g/dL) | 33.5 ± 0.4 | 33.1 ± 0.5 | 33.4 ± 0.4 | 33.3 ± 0.6 | |
| CHCM (g/dL) | 34.1 ± 0.2 | 34.0 ± 0.4 | 34.2 ± 0.3 | 34.1 ± 0.4 | |
| CH (pg) | 20.2 ± 0.4 | 20.3 ± 0.4 | 20.4 ± 0.7 | 20.0 ± 0.5 | |
| RDW (%) | 11.8 ± 0.5 | 12.0 ± 0.3 | 11.9 ± 0.4 | 12.1 ± 0.2 | |
| HDW (g/dL) | 2.20 ± 0.08 | 2.22 ± 0.08 | 2.28 ± 0.12 | 2.29 ± 0.09 | |
| Platelet (x10^3^/μL) | 1264 ± 221 | 1283 ± 239 | 1309 ± 50 | 1325 ± 141 | |
| MPV (fL) | 7.80 ± 0.2 | 7.66 ± 0.2 | 7.84 ± 0.4 | 7.58 ± 0.3 | |
| Retic (x10^3^/μL) | 269.8 ± 0.29 | 324.8 ± 18.9 ^*^ | 277.8 ± 39.6 | 307.4 ± 32.8 | |
| WBC (x10^3^/μL) | 7.83 ± 4.18 | 8.62 ± 1.70 | 4.95 ± 1.05 | 7.83 ± 3.64 | |
| Neutrophils (x10^3^/μL) | 1.30 ± 0.66 | 1.74 ± 0.68 | 0.84 ± 0.21 | 1.08 ± 0.46 |  |
| Lympocytes (x10^3^/μL) | 6.21 ± 3.72 | 6.64 ± 1.79 | 3.98 ± 0.96 | 6.50 ± 3.11 |  |
| Monocytes (x10^3^/μL) | 0.14 ± 0.08 | 0.12 ± 0.03 | 0.08 ± 0.02 | 0.13 ± 0.05 |  |
| Eosinophils (x10^3^/μL) | 0.09 ± 0.07 | 0.07 ± 0.08 | 0.03 ± 0.01 | 0.05 ± 0.04 |  |
| Basophils (x10^3^/μL) | 0.01 ± 0.02 | 0.02 ± 0.01 | 0.01 ± 0.01 | 0.01 ± 0.01 |  |
| LUC (x10^3^/μL) | 0.08 ± 0.09 | 0.03 ± 0.02 | 0.02 ± 0.01 | 0.06 ± 0.04 |  |

Values are presented as the mean ± SD. RBC, red blood cell; MCV, mean corpuscular volume; MCH, mean corpuscular hemoglobin; MCHC, mean corpuscular hemoglobin concentration; CHCM, cell hemoglobin concentration mean; CH, cell hemoglobin; RDW, red cell distribution width; HDW, hemoglobin distribution width; MPV, mean platelet volume; WBC, white blood cell; LUC, large unstained cell. ^a^Vehicle control group. ^*^ *P* < 0.05 vs vehicle control group.

**Table S10. Serum biochemical changes after repeated (2-week) administration of KMS88009 in rats.**

| Parameters | Dose (mg/kg/day) | | | |
| --- | --- | --- | --- | --- |
|  | 0 ^a^ | 100 | 300 | 1,000 |
| AST (IU/L) | 147.6 ± 28.9 | 152.3 ± 47.1 | 149.2 ± 24.3 | 162.0 ± 19.1 |
| ALT (IU/L) | 36.4 ± 4.6 | 37.3 ± 6.2 | 32.9 ± 4.7 | 32.7 ± 7.8 |
| ALP (IU/L) | 808.6 ± 103.0 | 723.4 ± 40.3 | 755.6 ± 165.7 | 736.0 ± 109.1 |
| T-Bil (mg/dL) | 0.04 ± 0.02 | 0.01 ± 0.01^*^ | 0.02 ± 0.01 | 0.02 ± 0.01 |
| Albumin (g/dL) | 2.42 ± 0.08 | 2.52 ± 0.18 | 2.4 ± 0.00 | 2.44 ± 0.05 |
| TP (g/dL) | 5.62 ± 0.13 | 5.66 ± 0.17 | 5.56 ± 0.23 | 5.62 ± 0.19 |
| A / G ratio | 0.75 ± 0.05 | 0.82 ± 0.08 | 0.77 ± 0.06 | 0.76 ± 0.04 |
| Glucose (mg/dL) | 130.9 ± 6.0 | 159.2 ± 23.5 | 148.0 ± 23.5 | 141.3 ± 15.6 |
| CHO (mg/dL) | 74.0 ± 9.0 | 79.4 ± 15.2 | 81.8 ± 11.6 | 80.4 ± 18.2 |
| TG (mg/dL) | 45.2 ± 29.5 | 47.0 ± 29.5 | 29.4 ± 7.2 | 30.8 ± 7.7 |
| LDH (IU/L) | 128.0 ± 40.5 | 168.0 ± 36.4 | 127.2 ± 47.0 | 126.0 ± 53.9 |
| CK (IU/L) | 261.2 ± 27.0 | 258.4 ± 54.3 | 222.0 ± 56.7 | 260.2 ± 62.0 |
| BUN (mg/dL) | 11.4 ± 2.2 | 12.2 ± 1.5 | 12.3 ± 2.8 | 11.9 ± 0.8 |
| CRE (mg/dL) | 0.4 ± 0.0 | 0.44 ± 0.05 | 0.42 ± 0.04 | 0.4 ± 0.00 |
| B / C ratio | 28.5 ± 5.5 | 28.3 ± 6.1 | 29.6 ± 8.1 | 29.7 ± 2.0 |
| Na^+^ (mmol/L) | 139.7 ± 0.9 | 140.5 ± 1.2 | 139.8 ± 1.8 | 140.1 ± 1.2 |
| K^+^ (mmol/L) | 4.63 ± 0.23 | 4.58 ± 0.43 | 4.73 ± 0.26 | 4.87 ± 0.22 |
| Cl^-^ (mmol/L) | 100.4 ± 2.6 | 99.6 ± 1.5 | 101.2 ± 1.2 | 101.3 ± 1.6 |

Values are presented as the mean ± SD. AST, aspartate aminotransferase; ALT, alanine aminotransferase; ALP, alkaline phosphatase, T-Bil, total bilirubin; TP, total protein; A / G, albumin-to-globulin; CHO, total cholesterol; TG, triglyceride; LDH, lactate dehydrogenase; CK, creatine kinase; BUN, blood urea nitrogen; CRE, creatinine; B / C, blood urea nitrogen-to-creatinine. ^a^Vehicle control group. ^*^ *P* < 0.05 vs vehicle control group.

**Table S11. Organ weights after repeated (2-week) administration of KMS88009 in rats.**

| Parameters | Dose (mg/kg/day) | | | |
| --- | --- | --- | --- | --- |
|  | 0 ^a^ | 100 | 300 | 1,000 |
| Body Weight (g) | 318.370 ± 5.583 | 316.188 ± 16.093 | 312.382 ± 14.169 | 319.932 ± 12.441 |
| Left adrenal (g) | 0.025 ± 0.003 | 0.027 ± 0.005 | 0.026 ± 0.004 | 0.025 ± 0.004 |
| Rate body weight / left adrenal (%) | 0.008 ± 0.001 | 0.009 ± 0.002 | 0.008 ± 0.001 | 0.008 ± 0.001 |
| Right adrenal (g) | 0.023 ± 0.004 | 0.025 ± 0.002 | 0.025 ± 0.004 | 0.026 ± 0.003 |
| Rate body weight / right adrenal (%) | 0.007 ± 0.001 | 0.008 ± 0.001 | 0.008 ± 0.001 | 0.008 ± 0.001 |
| Left kidney (g) | 1.259 ± 0.028 | 1.279 ± 0.083 | 1.237 ± 0.082 | 1.342 ± 0.067 |
| Rate body weight / left kidney (%) | 0.415 ± 0.012 | 0.404 ± 0.009 | 0.396 ± 0.020 | 0.420 ± 0.025 |
| Right kidney (g) | 1.331 ± 0.099 | 1.310 ± 0.101 | 1.266 ± 0.035 | 1.334 ± 0.067 |
| Rate body weight / right kidney (%) | 0.418 ± 0.016 | 0.414 ± 0.026 | 0.406 ± 0.008 | 0.418 ± 0.030 |
| Thymus (g) | 0.697 ± 0.132 | 0.649 ± 0.073 | 0.613 ± 0.115 | 0.621 ± 0.073 |
| Rate body weight / thymus (%) | 0.218 ± 0.034 | 0.205 ± 0.022 | 0.196 ± 0.036 | 0.194 ± 0.017 |
| Spleen (g) | 0.701 ± 0.055 | 0.731 ± 0.188 | 0.629 ± 0.130 | 0.749 ± 0.112 |
| Rate body weight / spleen (%) | 0.227 ± 0.029 | 0.230 ± 0.048 | 0.201 ± 0.037 | 0.234 ± 0.031 |
| Heart (g) | 1.069 ± 0.062 | 1.067 ± 0.098 | 1.002 ± 0.038 | 1.074 ± 0.065 |
| Rate body weight / heart (%) | 0.336 ± 0.012 | 0.337 ± 0.024 | 0.321 ± 0.011 | 0.336 ± 0.014 |
| Lung (g) | 1.256 ± 0.060 | 1.212 ± 0.077 | 1.214 ± 0.128 | 1.279 ± 0.094 |
| Rate body weight / lung (%) | 0.395 ± 0.023 | 0.384 ± 0.031 | 0.389 ± 0.037 | 0.400 ± 0.030 |
| Liver (g) | 10.061 ± 0.479 | 10.988 ± 0.881 | 10.601 ± 0.687 | 11.173 ± 0.202 |
| Rate body weight / liver (%) | 3.162 ± 0.110 | 3.473 ± 0.154 | 3.393 ± 0.133 | 3.495 ± 0.093 |
| Left testis (g) | 1.403 ± 0.159 | 1.440 ± 0.114 | 1.515 ± 0.189 | 1.366 ± 0.053 |
| Rate body weight / left testis (%) | 0.441 ± 0.048 | 0.456 ± 0.036 | 0.487 ± 0.075 | 0.427 ± 0.025 |
| Right testis (g) | 1.389 ± 0.147 | 1.450 ± 0.133 | 1.420 ± 0.086 | 1.365 ± 0.036 |
| Rate body weight / right testis (%) | 0.436 ± 0.042 | 0.459 ± 0.042 | 0.455 ± 0.023 | 0.427 ± 0.021 |
| Brain (g) | 1.933 ± 0.055 | 1.948 ± 0.032 | 1.680 ± 0.211^**^ | 1.922 ± 0.009 |
| Rate body weight / brain (%) | 0.608 ± 0.026 | 0.617 ± 0.026 | 0.537 ± 0.059 | 0.601 ± 0.024 |

Values are presented as the mean ± SD. ^a^Vehicle control group. ^**^ *P* < 0.01 vs vehicle control group.

**Table S12. Escape latency of hidden platform test in Morris water maze.**

|  | | **Wild Type (WT)** | **Transgenic (TG)** | | | | |
| --- | --- | --- | --- | --- | --- | --- | --- |
| **Scyllo-inosi.** | | **−** | **−** | **+++** | **−** | **−** | **−** |
| **KMS88009** | | **−** | **−** | **−** | **+** | **++** | **+++** |
|  | | **Latency (s)** | | | | | |
| Day 1 |  | 48.4 ± 2.4 | 55.4 ± 1.9 | 49.1 ± 2.4 | 50.5 ± 2.1 | 49.2 ± 2.4 | 48.3 ± 3.0 |
|  | vs. WT(-/-) | - | n.s. | n.s. | n.s. | n.s. | n.s. |
|  | vs. TG(-/-) | n.s. | - | n.s. | n.s. | n.s. | n.s. |
|  | vs. TG(+++/-) | n.s. | n.s. | - | n.s. | n.s. | n.s. |
| Day 2 |  | 35.6 ± 2.5 | 40.1 ± 2.6 | 43.1 ± 2.6 | 39.8 ± 2.7 | 38.3 ± 3.0 | 37.6 ± 5.2 |
|  | vs. WT(-/-) | - | n.s. | n.s. | n.s. | n.s. | n.s. |
|  | vs. TG(-/-) | n.s. | - | n.s. | n.s. | n.s. | n.s. |
|  | vs. TG(+++/-) | n.s. | n.s. | - | n.s. | n.s. | n.s. |
| Day 3 |  | 28.6 ± 3.1 | 44.0 ± 2.4 | 44.7 ± 4.2 | 31.1 ± 2.5 | 30.6 ± 3.3 | 34.3 ± 4.3 |
|  | vs. WT(-/-) | - | < 0.05^*^ | < 0.01^**^ | n.s. | n.s. | n.s. |
|  | vs. TG(-/-) | < 0.05^*^ | - | n.s. | < 0.05^*^ | n.s. | n.s. |
|  | vs. TG(+++/-) | n.s. | n.s. | - | < 0.05^*^ | n.s. | n.s. |
| Day 4 |  | 26.3 ± 2.4 | 42.5 ± 3.8 | 37.5 ± 4.0 | 35.1 ± 2.8 | 32.9 ± 3.0 | 32.0 ± 4.0 |
|  | vs. WT(-/-) | - | < 0.01^**^ | < 0.05^*^ | n.s. | n.s. | n.s. |
|  | vs. TG(-/-) | < 0.01^**^ | - | n.s. | n.s. | n.s. | n.s. |
|  | vs. TG(+++/-) | n.s. | n.s. | - | n.s. | n.s. | n.s. |
| Day 5 |  | 31.6 ± 3.2 | 43.6 ± 2.6 | 44.6 ± 3.3 | 41.8 ± 3.2 | 36.3 ± 4.2 | 29.6 ± 4.1 |
|  | vs. WT(-/-) | - | n.s. | n.s. | n.s. | n.s. | n.s. |
|  | vs. TG(-/-) | n.s. | - | n.s. | n.s. | n.s. | n.s. |
|  | vs. TG(+++/-) | < 0.05^*^ | n.s. | - | n.s. | n.s. | n.s. |
| Day 6 |  | 22.0 ± 1.7 | 46.1 ± 3.8 | 39.0 ± 4.3 | 32.9 ± 1.9 | 29.3 ± 3.9 | 33.4 ± 4.8 |
|  | vs. WT(-/-) | - | < 0.001^***^ | < 0.01^**^ | n.s. | n.s. | n.s. |
|  | vs. TG(-/-) | < 0.01^**^ | - | n.s. | < 0.05^*^ | < 0.05^*^ | n.s. |
|  | vs. TG(+++/-) | < 0.01^**^ | n.s.**^.^** | - | n.s. | n.s. | n.s. |
| Day 7 |  | 18.1 ± 1.8 | 42.0 ± 4.2 | 38.4 ± 4.4 | 31.7 ± 2.6 | 28.6 ± 3.5 | 30.0 ± 2.6 |
|  | vs. WT(-/-) | - | < 0.001^***^ | < 0.001^***^ | < 0.05^*^ | n.s. | n.s. |
|  | vs. TG(-/-) | < 0.001^***^ | - | n.s. | < 0.05^*^ | < 0.05^*^ | n.s. |
|  | vs. TG(+++/-) | < 0.001^***^ | n.s. | - | n.s. | n.s. | n.s. |
| Day 8 |  | 15.9 ± 1.5 | 41.6 ± 3.2 | 37.2 ± 4.3 | 26.6 ± 3.0 | 25.6 ± 3.0 | 27.9 ± 4.6 |
|  | vs. WT(-/-) | - | < 0.001^***^ | < 0.001^***^ | n.s. | n.s. | n.s. |
|  | vs. TG(-/-) | < 0.001^***^ | - | n.s. | < 0.05^*^ | < 0.05^*^ | n.s. |
|  | vs. TG(+++/-) | < 0.001^***^ | n.s. | - | n.s. | n.s. | n.s. |

The error bars represent the SEM. One-way ANOVA followed by Tukey post-test was performed in all statistical analyses. (^*^ *P* < 0.05, ^**^ *P* < 0.01, ^***^ *P* < 0.001, n.s.: no significance).
